# Relationship between acetaldehyde concentration in mouth air and characteristics of microbiota of tongue dorsum in Japanese healthy adults: a cross-sectional study

**DOI:** 10.1590/1678-7757-2018-0635

**Published:** 2019-06-13

**Authors:** Aya YOKOI, Daisuke EKUNI, Hironobu HATA, Mayu YAMANE-TAKEUCHI, Takayuki MARUYAMA, Reiko YAMANAKA, Manabu MORITA

**Affiliations:** 1 Okayama University Graduate School of Medicine, Dentistry and Pharmaceutical Sciences, Department of Preventive Dentistry, Okayama, Japan.; 2 Okayama University, Dental School, Advanced Research Center for Oral and Craniofacial Sciences, Okayama, Japan.; 3 National Hospital Organization Hokkaido Cancer Center, Department of Dentistry and Oral Surgery, Sapporo, Japan.; 4 Okayama University Hospital, Center for Innovative Clinical Medicine, Okayama, Japan.; 5 Okayama University Hospital, Division of Hospital Dentistry, Central Clinical Department, Okayama, Japan.

**Keywords:** Acetaldehyde, Tongue, Microbiota, Sequence analysis, Cross-sectional studies

## Abstract

Acetaldehyde, associated with consumption of alcoholic beverages, is known to be a carcinogen and to be related to the tongue dorsum.

**Objective:**

The aim of this study was to investigate the relationship between acetaldehyde concentration in mouth air and bacterial characteristics on the tongue dorsum.

**Methodology:**

Thirty-nine healthy volunteers participated in the study. Acetaldehyde concentrations in mouth air were evaluated by a high-sensitivity semiconductor gas sensor. A 16S rRNA gene sequencing technique was used to compare microbiomes between two groups, focusing on the six samples with the highest acetaldehyde concentrations (HG) and the six samples with lowest acetaldehyde concentrations (LG).

**Results:**

Acetaldehyde concentration increased in correlation with the increase in bacterial count (p=0.048). The number of species observed in the oral microbiome of the HG was higher than that in the oral microbiome of the LG (p=0.011). The relative abundances of *Gemella sanguinis*, *Veillonella parvula* and *Neisseria flavescens* in the oral microbiome of the HG were higher than those in the oral microbiome of the LG (p<0.05).

**Conclusion:**

Acetaldehyde concentration in mouth air was associated with bacterial count, diversity of microbiome, and relative abundance of *G. sanguinis*, *V. parvula*, and *N. flavescens*.

## Introduction

Acetaldehyde, associated with consumption of alcoholic beverages, is known as a gaseous carcinogen leading to oral, esophageal, and gastrointestinal tract cancers.^1^ ^-^ ^3^ The 2012 Monograph of the International Agency for Research on Cancer concluded that “alcohol consumption is carcinogenic to humans; ethanol in alcoholic beverages is carcinogenic to humans; and acetaldehyde associated with the consumption of alcoholic beverages is carcinogenic to humans”.^4^ Previous studies have shown that acetaldehyde causes point mutations in DNA and formation of DNA adducts, inducing sister chromatid exchange and gross chromosomal aberrations.^5^ ^-^ ^7^

Acetaldehyde can be generated in the human oral cavity by microorganisms such as yeasts and bacteria. For example, studies demonstrated that oral *Candida* species are capable of producing significant amounts of acetaldehyde from ethanol and glucose *in vitro*.^8^ In addition, *Neisseria* and *Streptococcus* species in saliva samples have been investigated regarding their role in acetaldehyde production.^9^ However, the relationship between microbiome and physiological acetaldehyde concentration in mouth air is uncertain. It is important to identify the microbial factors related to acetaldehyde accumulation in mouth air, because individuals who have oral microbiome with high ability of producing acetaldehyde could have a high risk of cancer by consumption of alcoholic beverages.

A previous study revealed that acetaldehyde concentration in mouth air is associated with the tongue coating,^10^ which may be a source of local production of this compound by the oral microbiome. Acetaldehyde concentration in healthy adults with a tongue-coating-status score of 3 (i.e., coating covering more than two-thirds of the tongue dorsum surface) was significantly higher than in healthy adults with a score of 0/1 (i.e., no coating or coating covering less than one-third of the tongue dorsum surface, respectively).^10^ The tongue coating consists of bacteria, large quantities of desquamated epithelial cells, blood metabolites, different kinds of food remnants, and leucocytes derived from periodontal pockets.^11^ ^,^ ^12^ However, little is known about which organisms of the tongue coating microbiome affect the acetaldehyde concentration in mouth air.

The hypothesis was that the acetaldehyde concentration in mouth air is related to the presence of certain organisms of the microbiome on the tongue. Therefore, the aim of this study was to investigate the relationship between characteristics of the microbiome on the tongue dorsum and acetaldehyde concentration in mouth air in healthy adults.

## Methodology

### Ethics statement

This is a cross-sectional study. The Ethics Committee of the Okayama University Graduate School of Medicine, Dentistry and Pharmaceutical Sciences approved the study protocol (KEN1506-074). All procedures were in accordance with the ethical standards of the responsible committees on human experimentation (institutional and national) and with the Helsinki Declaration of 1964 and later versions. Written informed consent was obtained from all participants.

### Participants

Thirty-nine healthy subjects (12 males and 27 females, ranging in age from 20 to 30 years old) who attended the Dental Clinic of Okayama University Hospital were enrolled as voluntary participants in this study. The recruitment period was from October 2014 to November 2015. Enrollment criteria excluded subjects with respiratory, digestive system, otorhinolaryngologic, or liver diseases, as well as subjects taking any antibiotic or undergoing other antimicrobial therapy.

### Measurement of acetaldehyde

The Sensor Gas Chromatograph SGEA-P2 (FIS Inc., Itami, Japan) was used to measure acetaldehyde concentrations in mouth air.^10^ This system uses ambient air as a carrier gas, therefore a high-pressure gas cylinder is not necessary. Participants were advised to abstain from food or drink and to refrain from their standard oral hygiene practice on the morning of the day of measurement.^10^ Participants also were instructed to refrain from eating strong-smelling foods for at least 48 h, from using perfumes for 24 h, from smoking for 24 h, and from drinking alcohol for 12 h prior to measurement.

Actual measurements were conducted in the morning, between 8:00 and 9:00 am. Participants kept their mouths closed for three minutes prior to measurement. As acetaldehyde is highly volatile, air contamination in the oral cavity was avoided as much as possible. Notably, during collection of mouth air (using a syringe), each participant breathed through the nose. Immediately upon collection of the sample gas into the syringe, the sample was injected and parameters were measured.^10^ ^,^ ^13^ An injection of the sample gas (5 mL) from the syringe into the detector initiated the measurement automatically. Measurement was completed in eight minutes.

To assess the reproducibility of the sampling, the two-days experiment was set for calibration. Each measurement was performed in duplicate. In the intra- and inter- assay, the error was below 5%.

To assess the reproducibility of the measurement, defined samples containing 100-10,000 ppb acetaldehyde were used for calibration. Each measurement was performed in duplicate. Both intra- and inter- assay coefficients of variation were below 5%.

### Oral examination

Status of tongue coating was assessed according to its distribution area, with scoring as follows: 0, none visible; 1, less than one-third of the tongue dorsum surface covered; 2: less than two-thirds of the tongue dorsum surface covered; 3: more than two-thirds of the tongue dorsum surface covered.^14^

All clinical procedures were performed by one of four trained dentists (A.Y., M. Y., T. M., R. Y., and D. E.). Scoring among the dentists was calibrated by confirming that these dentists showed good intra- and inter-examiner agreement for the examination, as evaluated by kappa statistics over 0.8.

### Measurement of total bacterial counts

A rapid bacterial detection apparatus (PHC Holdings Co., Ltd., Tokyo, Japan) that consisted of the elements necessary for dielectrophoretic impedance measurement (DEPIM) was used.^15^ ^,^ ^16^ These elements included an electrode chip to capture bacteria, a cell-retaining sample solution, an alternating current circuit for dielectrophoresis, and an impedance measurement circuit. Measurement was initiated by placing the sample solution (approximately 5 mL) and electrode chip inside the device and pressing a button. The results of measurement then were shown on a liquid crystal display. The sample was collected from the median area of the tongue dorsum using a swab (men-tip^*®*^; J.C.B. Industry Limited, Tokyo, Japan).^15^ The collection pressure was about 21 g, and a 1-cm span was sampled by rubbing the swab back and forth three times.^16^

To assess the reproducibility of the sampling, the measurement was performed in duplicate at the same time. In the intra-assay, the error was below 5%.

### Detection of *Candida* species

Acetaldehyde can be generated in the human oral cavity by microorganisms such as *Candida* and bacteria^8^. We detected *Candida* and bacteria separately. We used CHROMagar Candida medium (CHROMagar Candida, Kanto Chemical Co., Inc., Tokyo, Japan) (pH 6.1) to detect *Candida albicans*, *Candida tropicalis*, and *Candida krusei*. The medium comprised (*per* liter) peptone (10 g), glucose (20 g), agar (15 g), chloramphenicol (0.5 g), and Chromogenic IX (2 g), and was prepared according to the manufacturer’s instructions. All samples swabbed from the oral mucosa and tongue were plated on the medium and then permitted to grow for 48 hours at 37°C. The color and morphology of the resulting colonies were recorded, and the organism classification was based on comparison to photographic images, including green colonies for *C. albicans*, steel blue colonies for *C. tropicalis*, and rose-colored colonies for *C. krusei*.^17^

### Assessment of alcohol sensitivity

Alcohol sensitivity, which can reflect acetaldehyde production in the human body,^18^ was assessed by inferring the acetaldehyde dehydrogenase (ALDH) genotype of each participant. The ethanol patch test (ASK Human Care, Co., Ltd., Tokyo, Japan) was used to infer the ALDH genotype of each participant.^19^ Briefly, a plaster patch impregnated with ethanol was fixed on adhesive tape. The plaster patch was attached to the inner surface of the arm for 20 minutes and removed according to the manufacturer’s procedure. Patients whose patch area exhibited erythema after plaster removal were judged to be positive for reaction to alcohol and inferred to have the ALDH genotype (ALDH2*1/*2 or *2/*2). If negative, the participants were assigned to the ALDH genotype (ALDH2*1/*1). Then, alcohol sensitivity was characterized as “high” (ALDH2*1/*2 or *2/*2) or ‘low’ (ALDH2*1/*1).

### Questionnaire

In addition to age, sex, and general condition, the questionnaire covered the items ‘smoking habits’ and ‘alcohol consumption’. Smoking status was characterized as “never”, “past”, and “current”.^20^ Information regarding alcohol consumption was characterized as “never”, “light” (less than five days *per* week), “moderate” (five or more days *per* week, less than twice a day), and “heavy” (five or more days *per* week, more than twice a day).^21^

### Sample collection for identification of bacteria on tongue dorsum

Microbiome samples from 12 subjects were collected after measurement of acetaldehyde concentration in the mouth air and other examinations. Specifically, samples of the microbiome from the tongue dorsum (tongue coating) were collected from six individuals, each with the highest (high group; HG) and lowest (low group; LG) acetaldehyde concentrations in the mouth air. These samples were collected between 11:00 am and 12:00 pm. Each sample was collected from the median area of the tongue dorsum using a swab (men-tip^*®*^) that had been previously wetted by immersion in 5 mL of pure water and then rolled over the sampled surface using moderate pressure (21 g) and circular motion.^22^ The 12 samples then were extracted (as described below) to permit investigation of the characteristics of the respective microbiomes.

### Identification of bacteria on tongue dorsum

The characteristics of the oral microbiomes from the HG and LG were compared by focusing on the relative abundance of bacteria that had been previously reported^9^ to produce acetaldehyde. Microbial DNA was extracted from each swab sample using a QIAamp DNA Mini and Blood Kit (QIAGEN, Hilden, Germany) according to the manufacturer’s instructions. The V3/V4 region of the 16S rRNA genes were amplified with primers 357F and 781R, and sequenced on an Illumina MiSeq instrument (MiSeq Reagent V3 600 cycles; Illumina, San Diego, USA) at Okayama University Hospital Biobank (Okayama University Hospital, Okayama, Japan). Raw sequence data was screened, trimmed, and filtered, and the noise, barcodes, and chimeric sequences were depleted from the dataset using USEARCH version 8.0.1623^23^, FastQC version 11.3 (http://www.bioinformatics.babraham.ac.uk/projects/fastqc/ – publicly available software), and QIIME version 1.9.1 at Oral Microbiome Center (Taniguchi Dental Clinic, Takamatsu, Japan).^24^ Sequences shorter than 400 bases were excluded from analysis. Operational taxonomic units (OTUs), defined by a 97% similarity to database sequences, were picked using the UCLUST algorithm. All sequences were queried against a BLAST database containing oral bacterial 16S rRNA gene sequences in the HOMD (Human Oral Microbiome Database 16S rRNA RefSeq version 14.51).^25^ Values for α and β diversity were evaluated through QIIME. The α diversity was assessed using numbers of observed species. The β diversity was assessed using weighted UniFrac distance matrices accounting for both presence or absence of observed organisms and abundances.^26^

### Statistical analysis

Sample size was estimated by comparison with the diversity of the nasal microbiome determined in a previous study.^27^ Based on the data, minimum sample sizes were defined as consisting of at least five individuals in order to provide a power of 95% with an alpha of 0.05 using the Mann-Whitney *U* test. We calculated the sample size by using nQuery variances (nQuery Advisor 6.01, Boston, U.S.A).^28^ Data analysis was performed using the Statistical Package for the Social Sciences (SPSS version 20, IBM Japan, Tokyo, Japan). There were no missing data.

The Mann-Whitney *U* test was used to compare acetaldehyde concentrations in mouth air between groups, specifically: male vs. female, low- vs. high-alcohol sensitivity, or never vs. light and moderate (for alcohol consumption). The differences in parameters among the three tongue coating groups (scores 0–1, 2, and 3) were analyzed by the Mann-Whitney *U* test with the Bonferroni correction. Because there were no participants without tongue coating (tongue coating score 0), those with scores of 0 and 1 were considered as a single class. The level of significance was set at p<0.017 according to the Bonferroni correction. The association between acetaldehyde concentration and other parameters was analyzed using the Spearman’s correlation coefficient, with a significance level of p<0.05.

For comparison of bacterial communities in HG and LG, the phylogeny-based weighted UniFrac distance metric was used and principal coordinate analysis (PCoA) plots were generated with QIIME version 1.9.1.^26^ The ANOSIM (Analysis of Similarity) test was performed using the Vegan packages in the R version 3.5.1 software. ANOSIM was performed on the weighted UniFrac distance metrics to detect significance in microbial communities between the two groups (HG *vs.* LG). The Mann-Whitney *U* test was used to analyze the diversity of the microbiome on the tongue. As noted above, we selected the bacteria that previously^9^ had been shown to be capable of producing acetaldehyde. The Mann-Whitney *U* test was used to analyze the significance of differences in the relative abundances of bacteria between the microbiomes of the two groups.

## Results

Thirty-nine subjects (12 males and 27 females; 20–30 years old) completed this study. Table 1 shows the characteristics of the study participants. More than half of the patients were female. There were no *Candida* carriers or current smokers. We measured the acetaldehyde concentration in the mouth air from each participant. Acetaldehyde concentration in mouth air was 146.5 (73.4, 237.0) ppb [median (25%, 75%)].

**Table 1:** Characteristics of study participants (n=39)

Variable		Median (25%, 75%)/Number (%)		
		Total (n=39)	HG (n=6)	LG (n=6)
Acetaldehyde concentration (ppb)		146.5 (73.4, 237.0)	292.7(243.8, 441.6)	117.7 (97.3, 139.7)
Age (years)		21 (21, 23)	21 (21, 21)	22 (20, 23)
Sex	Male	12 (30.8)	0 (0.0)	4 (66.6)
Tongue coating status (score)	0	0 (0.0)	0 (0.0)	0 (0.0)
	1	5 (12.8)	0 (0.0)	1 (16.7)
	2	14 (35.9)	1 (16.7)	3 (50.0)
	3	20 (51.3)	5 (83.3)	2 (33.3)
Bacterial count × 107 (CFUs/mL)		1.15 (0.35, 1.68)	0.43 (0.38, 1.51)	0.33 (0.24, 1.21)
Candida species	+	0 (0.0)	0 (0.0)	0 (0.0)
Alcohol sensitivity	Low	23 (59.0)	3 (50.0)	3 (50.0)
Smoking status	Never	38 (97.4)	6 (100.0)	6 (100.0)
	Past	1 (2.6)	0 (0.0)	0 (0.0)
	Current	0 (0.0)	0 (0.0)	0 (0.0)
Alcohol consumption	Never	20 (51.3)	3 (50.0)	3 (50.0)
	Light	18 (46.2)	3 (50.0)	3 (50.0)
	Moderate	1 (2.6)	0 (0.0)	0 (0.0)
	Heavy	0 (0.0)	0 (0.0)	0 (0.0)

Acetaldehyde concentration in participants with a tongue coating status score of 3 was significantly higher than that in participants with a score of 0/1 [215.4 (96.1, 270.1) vs. 48.3 (33.6, 56.7)] (p<0.001) (Table 2).

**Table 2:** Difference in acetaldehyde concentration in mouth air

Variable		Acetaldehyde concentration (ppb)
Sex	Male	141.7 (112.7, 221.3)*
	Female	147.2 (58.3, 254.6)
Tongue coating status score	0/1	48.3 (33.6, 56.7)
	2	153.6 (71.4, 205.8)
	3	215.4 (96.1, 270.1)^†^
Alcohol sensitivity	Low	136.8 (73.4, 223.0)
	High	185.5 (86.4, 264.6)
Alcohol consumption	Never	147.2 (58.2, 240.5)
	Light/Moderate	136.8 (92.7, 237.0)

* Median (25%, 75%)

^†^ p<0.017, compared to the 0/1 group (tongue coating status), Mann-Whitney *U* test with Bonferroni correction

There was a significant correlation between the bacterial count measured using the DEPIM method and acetaldehyde concentration in mouth air (Table 3) (p=0.048). However, age had no association with acetaldehyde concentration (Table 3).

**Table 3:** Correlation between acetaldehyde concentration (in mouth air) and other parameters

Variable	ρ	p-value
Age	0.107	0.516*
Bacterial count	0.319	0.048

* p<0.05, Spearman’s correlation coefficients

**Figure 1:**
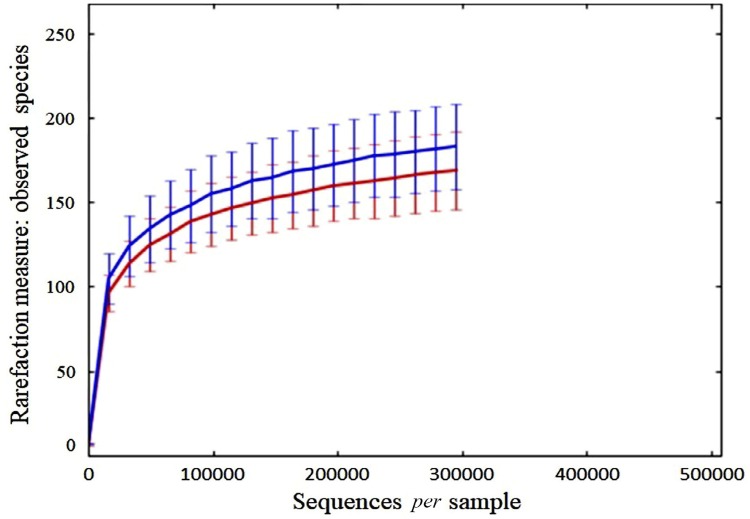
Rarefaction analysis of 16S rRNA gene sequences obtained from samples. HG samples are in blue and LG samples are in red. Data are presented as median with error bars representing 25th and 75th percentiles (n=6/group)

Figure 1 shows the alpha rarefaction curve comparing species between HG and LG. The number of observed species in the tongue microbiome of the HG was significantly larger than that in the tongue microbiome of the LG (p=0.011).

**Figure 2:**
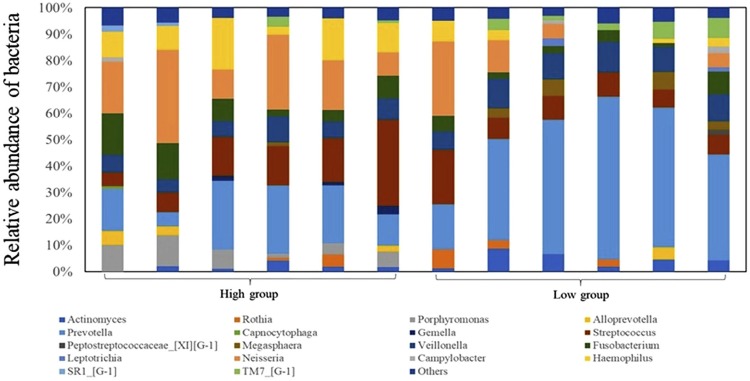
Column plots of genera and relative abundance of bacteria in tongue coating. Bacterial flora with relative abundance <1% were classified as “Others”

Figure 2 shows the relative abundance of bacterial genera in the tongue coating. The predominant genus in the tongue microbiomes of the HG and LG were *Neisseria* and *Prevotella*, respectively.

**Figure 3:**
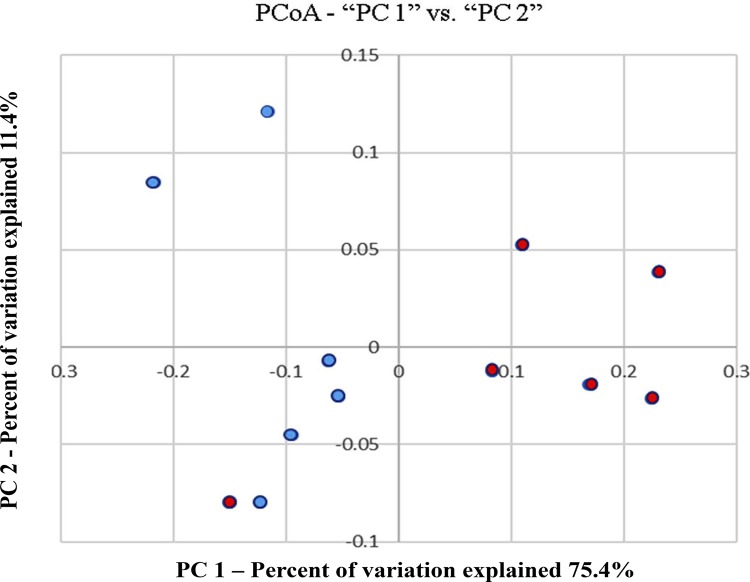
Principal coordinate analysis (PCoA) of weighted UniFrac distances of 16S rRNA genes. Data were obtained from HG (blue dots) and LG (red dots) samples (n=6/group)

The PCoA plot (Figure 3) revealed that the bacterial species in the HG microbiome clustered separately from those in the LG microbiome, except for one of the subjects in the LG group. ANOSIM of the weighted UniFrac distances showed that this difference in clustering was significant (R=0.5556, p=0.013).

**Figure 4:**
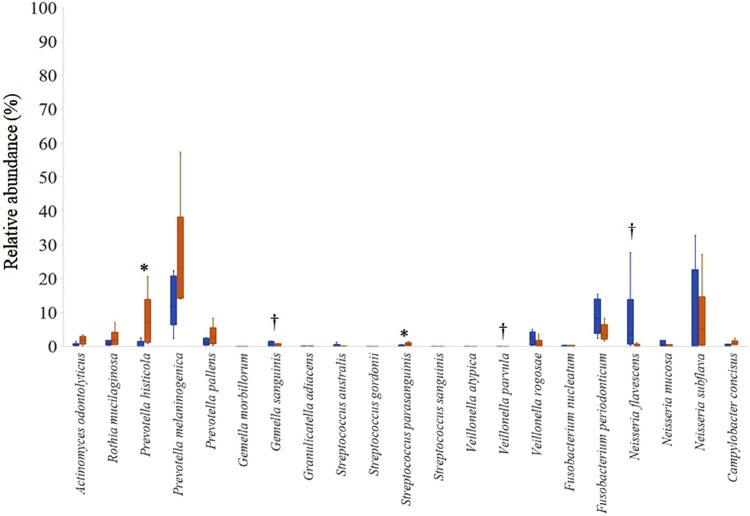
Difference in relative abundance of tongue microbiome between HG and LG. Data were presented as median with error bars representing the 25th and 75th percentiles (n=6/group). These were significant differences in relative abundance of some species between HG (blue bar) and LG (red bar)

Figure 4 shows the relative abundance of acetaldehyde-producing bacteria among the total bacteria. The relative abundances of *Gemella sanguinis, Veillonella parvula*, and *Neisseria flavescens* in the tongue microbiome of the HG were significantly greater than those in the tongue microbiome of the LG (p<0.05). In contrast, the relative abundances of *Prevotella histicola* and *Streptococcus parasanguinis* in the tongue microbiome of the HG were significantly smaller than those in the tongue microbiome of the LG (p<0.05).

* p<0.05, LG>HG; † p<0.05, HG>LG (Mann-Whitney *U* test)

## Discussion

In this study, the acetaldehyde concentration was positively associated with bacterial count on the tongue; diversity of the tongue microbiome; and relative abundance of *G. sanguinis, V. parvula*, and *N. flavescens*, species known to produce acetaldehyde. To the best of our knowledge, this is the first report to clarify the relationship between acetaldehyde concentration in mouth air and the characteristics of the microbiome on the tongue dorsum in healthy adults.

The focus was on the oral microbiome in the tongue dorsum because the major source of acetaldehyde production in mouth air is tongue coating.^10^ The tongue is the largest bacterial reservoir in the oral cavity and the tongue microbiome is a dominant source of salivary bacteria.^29^ ^-^ ^33^ This study considered that the characteristics of tongue microbiome is a representative of oral microbiome and the effects of other bacterial species reside in other parts of the oral cavity where aldehyde production may be small.

There was a significant correlation between acetaldehyde concentration in mouth air and the diversity of the tongue microbiome. In dental plaque, highly diverse communities result from the altered ecological conditions within the biofilm, because prolonged plaque accumulation contributes to the multiplication of attached bacteria.^34^ Acetaldehyde concentration is associated with tongue coating volume.^10^ Development of the tongue coating may result in an enhanced diversity of the tongue microbiome, thereby contributing to increased production of acetaldehyde in the mouth air.

There was a significant correlation between acetaldehyde concentration in mouth air and the number of CFUs on the tongue. This finding suggests that the bacteria on the tongue dorsum may be a source of local production of acetaldehyde; a decrease in the bacterial count on the tongue dorsum therefore would lead to a decreased concentration of acetaldehyde in the mouth air. Mechanical cleaning of the tongue with a specific tongue cleaner can reduce the tongue coating, average amount of total bacteria, and acetaldehyde concentration.^10^ ^,^ ^35^ Especially, the previous study reported that median acetaldehyde concentration decreased significantly after tongue cleaning from 222.0 to 141.9 ppb.^10^ Acetaldehyde, associated with consumption of alcoholic beverages, is a carcinogen that can induce oral, esophageal, and gastrointestinal tract cancers.^1^ ^-^ ^3^ Thus, tongue cleaning may decrease the risk of oral cancer in alcohol drinkers by decreasing the production of acetaldehyde by oral bacteria.

ANOSIM of the weighted UniFrac distances showed a significant difference between the HG and LG microbiomes (R=0.5556, p=0.013). The differences in the microbiomes on tongue dorsum may lead to the differences in acetaldehyde concentrations in mouth air. The relative abundances of *N. flavescens* and *G. sanguinis* in the HG microbiomes were significantly higher than those in the LG microbiomes. In contrast, the relative abundances of *P. histicola* and *S. parasanguinis* in the HG microbiomes were significantly lower than those in the LG microbiomes. However, a recent study reported results that differed from those of the present study. In that study, participants with type-I oral microbial communities (defined as those consisting specifically of *P. histicola* and *S. parasanguinis*) showed higher acetaldehyde production than those with the type-II oral microbial communities (defined as those consisting specifically of *N. flavescens* and *G. sanguinis*).^36^ These apparently contradictory findings may reflect differences in experimental conditions and patient characteristics. Notably, Yokoyama, et al.^36^ (2018) measured acetaldehyde production from saliva (which included the microbiome) when ethanol was added experimentally, as part of the assay; in contrast, the present study investigated the microbiome on the tongue and measured acetaldehyde concentrations in mouth air in the absence of extraneous ethanol.

The relative abundances of *N. flavescens* in the microbiomes of the HG were significantly higher than those in the microbiomes of the LG. A previous study reported that *N. flavescens* exhibited higher acetaldehyde production capacity than other bacteria that can generate acetaldehyde.^9^ *Neisseria* species are associated with oral lichen planus, which is known to be a precancerous condition.^37^ *N. flavescens* on the tongue may contribute to the incidence of oral cancer, through the higher production of acetaldehyde.

The participants in this study may be representative of the general population. First, the median bacterial count was 1.15×10^7^ CFUs/mL. This value was within the range (9.5×10^6^–2.9×10^7^ CFUs/mL) defined (using the same device) in a previous study of Japanese perioperative patients.^38^ Second, the median (25%, 75%) acetaldehyde concentration in mouth air was 146.5 (73.4, 237.0) ppb. These values were similar to those obtained in a previous study [170.7 (73.5, 306.3) ppb].^10^

This study has several limitations. First, all subjects were recruited at a single location (Okayama University), and the number of subjects and sampling area were small. Additional large-scale studies and sampling from various areas assessing the tongue are expected to provide information beyond the findings presented here. Second, the participants were healthy adults. In future studies, the relationship between acetaldehyde concentration and the bacterial count on the tongue or characteristics of microbiome among patients with cancer should be investigated. Third, dietary habits were not investigated, which might affect acetaldehyde concentration. However, participants skipped breakfast at the measurement day and refrained from eating strong-smelling foods for at least 48 h. Thus, the effects on acetaldehyde concentration might be few. Finally, this study was a cross-sectional study. A prospective cohort study, notably one that follows participants until the onset of cancer, would facilitate definition of the possible relationships among acetaldehyde concentrations, constituents of the oral microbial community, and cancer risk.

## Conclusions

This study revealed that acetaldehyde concentration in mouth air was positively associated with bacterial count; diversity of the microbiome; and the relative abundance of *G. sanguinis*, *V. parvula*, and *N. flavescens*, species that are known to be capable of producing acetaldehyde.
